# 2-Bromo-2-methyl-*N*-(4-nitro­phen­yl)propanamide

**DOI:** 10.1107/S1600536811005320

**Published:** 2011-02-19

**Authors:** Rodolfo Moreno-Fuquen, David E. Quintero, Fabio Zuluaga, Roberto L. A. Haiduke, Alan R. Kennedy

**Affiliations:** a Departamento de Química, Facultad de Ciencias, Universidad del Valle, Apartado 25360, Santiago de Cali, Colombia; b Instituto de Química, IQSC, Universidade de São Paulo, São Carlos, Brazil; c WestCHEM, Department of Pure and Applied Chemistry, University of Strathclyde, 295 Cathedral Street, Glasgow G1 1XL, Scotland

## Abstract

The title compound, C_10_H_11_BrN_2_O_3_, exhibits a small twist between the amide residue and benzene ring [the C—N—C—C torsion angle = 12.7 (4)°]. The crystal structure is stabilized by weak N—H⋯O, C—H⋯Br and C—H⋯O inter­actions. These lead to supra­molecular layers in the *bc* plane.

## Related literature

For initiators in ATRP (polymerization by atom transfer radical) processes, see: Matyjaszewski & Xia (2001 ▶); Pietrasik & Tsarevsky (2010 ▶). For graph-set notation of hydrogen-bond patterns, see: Etter (1990 ▶).
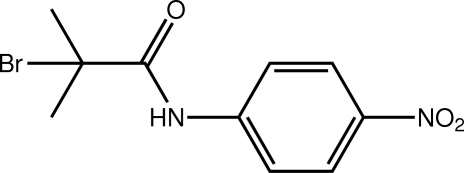


## Experimental

### 

#### Crystal data

C_10_H_11_BrN_2_O_3_
*M* *_r_* = 287.11
Monoclinic, 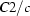

*a* = 24.1245 (12) Å
*b* = 5.8507 (3) Å
*c* = 15.4723 (8) Å
β = 91.837 (5)°
*V* = 2182.72 (19) Å^3^
*Z* = 8
Mo *K*α radiation
μ = 3.76 mm^−1^
*T* = 123 K
0.60 × 0.05 × 0.05 mm

#### Data collection

Oxford Diffraction Xcalibur E diffractometer
Absorption correction: analytical (*CrysAlis PRO*; Oxford Diffraction, 2009 ▶) *T*_min_ = 0.444, *T*_max_ = 1.000
5100 measured reflections
2633 independent reflections
2197 reflections with *I* > 2σ(*I*)
*R*_int_ = 0.032

#### Refinement

*R*[*F* ^2^ > 2σ(*F* ^2^)] = 0.032
*wR*(*F* ^2^) = 0.068
*S* = 1.06
2633 reflections
151 parameters
H atoms treated by a mixture of independent and constrained refinement
Δρ_max_ = 0.55 e Å^−3^
Δρ_min_ = −0.60 e Å^−3^

### 

Data collection: *CrysAlis CCD* (Oxford Diffraction, 2009 ▶); cell refinement: *CrysAlis CCD*; data reduction: *CrysAlis CCD*; program(s) used to solve structure: *SHELXS97* (Sheldrick, 2008 ▶); program(s) used to refine structure: *SHELXL97* (Sheldrick, 2008 ▶); molecular graphics: *ORTEP-3 for Windows* (Farrugia, 1997 ▶) and *Mercury* (Macrae *et al.*, 2006 ▶); software used to prepare material for publication: *SHELXL97*.

## Supplementary Material

Crystal structure: contains datablocks I, global. DOI: 10.1107/S1600536811005320/tk2720sup1.cif

Structure factors: contains datablocks I. DOI: 10.1107/S1600536811005320/tk2720Isup2.hkl

Additional supplementary materials:  crystallographic information; 3D view; checkCIF report

## Figures and Tables

**Table 1:** Hydrogen-bond geometry (Å, °)

*D*—H⋯*A*	*D*—H	H⋯*A*	*D*⋯*A*	*D*—H⋯*A*
C6—H6⋯O1	0.95	2.27	2.862 (3)	120
C7—H7⋯O1^i^	0.95	2.45	3.139 (3)	129
N1—H1n⋯O3^ii^	0.84 (3)	2.66 (3)	3.316 (3)	136 (2)
C10—H10⋯Br1^iii^	0.95	2.91	3.812 (2)	160

Symmetry codes: (i) 
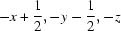
; (ii) 
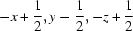
; (iii) 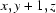.

**Fig. 1.**
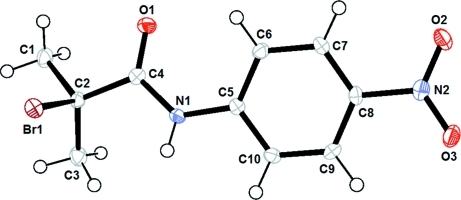
An ORTEP-3 (Farrugia, 1997) plot of (I) with the atomic labelling scheme. Displacement ellipsoids are drawn at the 50% probability level. H atoms are shown as spheres of arbitrary radius.

**Fig. 2.**
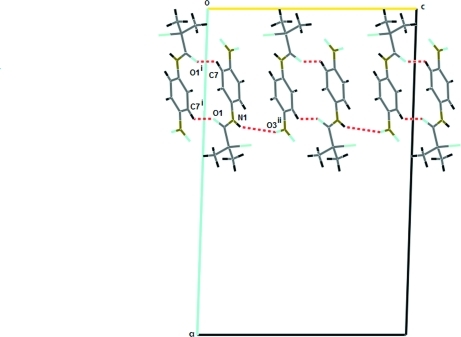
Part of the crystal structure of (I), showing the formation of R22(10) and R44(18) rings, running along [110]. Symmetry code: (i) -x+1,-y+1,-z; (ii) -x+2,-y+2,-z.

**Fig. 3.**
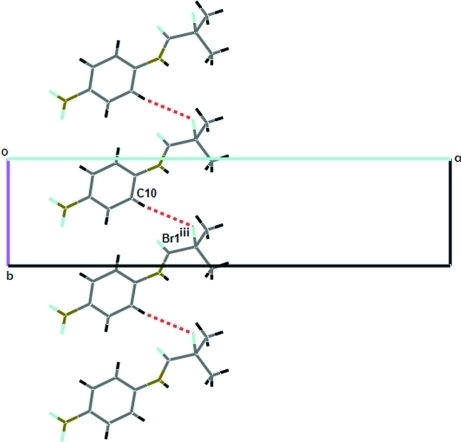
Part of the crystal structure of (I), showing the formation of an infinite zig-zag chain of dimers along [001] direction. Symmetry code: (iii) x,-y+1/2+1,+z-1/2.

## supplementary crystallographic information

### Comment

The title compound, (I), forms a part of a synthetic programme that the Polymer Research Group of Universidad del Valle is pursuing in order to obtain compounds that act as initiators of reactions in polymerization processes. Compound (I), Fig. 1, belongs to a series of polymeric ATRP (polymerization by atom transfer radical) initiators (Pietrasik & Tsarevsky, 2010); most initiators for ATRP processes are alkyl halides (Matyjaszewski & Xia, 2001). The C4—N1—C5—C6 torsion angle is 12.7 (4)°, indicating a small twist between the benzene ring and the amide. An intramolecular C—H···O interaction is observed (see Table 1).

In the crystal structure, weak C—H···O and N—H···O intermolecular interactions are detected (Table 1). In a first substructure (Fig. 2), molecules are linked by C7—H···O1^i^ contacts (i: -x+1/2,-y-1/2,-z) which leads to the formation of dimers with graphs-set notation, R^2^_2_(14) (Etter, 1990). In turn, N—H···O interactions link the dimers running along the *c* axis. The N1 atom acts as hydrogen bond donor to O3^ii^ (ii: -x+1/2,+y-1/2,-z+1/2). In a second substructure, C10—H···Br (Table 1) interactions provide further links between the dimers. Overall, the crystal structure comprises layers in the *bc* plane (Fig. 3).

### Experimental

The initial reagents were purchased from Aldrich Chemical Co. and were used as received. In a 100 mL round bottom flask 4-nitroaniline (3.258 mmol, 0.450 g), triethylamine (0.653 mmol, 0.066 g) were mixed, then a solution of 2-bromo isobuturyl bromide (0.704 g) in anhydrous THF (5 mL) was added drop wise, under an argon stream. The reaction was carried out in a dry bag overnight under magnetic stirring. The solid was filtered off and dichloromethane (20 mL) added to the organic phase which was washed with brine (40 mL) followed by water (10 mL). The solution was concentrated at low pressure affording colourless crystals and recrystalized from a solution of hexane and ethyl acetate (80:20). *M*. pt. 356 (1) K.

### Refinement

The H-atoms were placed geometrically [C—H = 0.95 Å for aromatic & C—H = 0.98 Å for methyl], refined in the riding model approximation with U_iso_(H) = 1.2–1.5U_eq_(C). The N-bound H atom was refined freely.

### Crystal data

**Table:**

C_10_H_11_BrN_2_O_3_	*F*(000) = 1152
*M**_r_* = 287.11	*D*_x_ = 1.747 Mg m^−^^3^
Monoclinic, *C*2/*c*	Melting point: 385(1) K
Hall symbol: -C 2yc	Mo *K*α radiation, λ = 0.71073 Å
*a* = 24.1245 (12) Å	Cell parameters from 2722 reflections
*b* = 5.8507 (3) Å	θ = 3.2–29.3°
*c* = 15.4723 (8) Å	µ = 3.76 mm^−^^1^
β = 91.837 (5)°	*T* = 123 K
*V* = 2182.72 (19) Å^3^	Fragment cut from needle, colourless
*Z* = 8	0.60 × 0.05 × 0.05 mm

### Data collection

**Table:**

Oxford Diffraction Xcalibur E diffractometer	2633 independent reflections
Radiation source: fine-focus sealed tube	2197 reflections with *I* > 2σ(*I*)
graphite	*R*_int_ = 0.032
ω scans	θ_max_ = 29.0°, θ_min_ = 3.2°
Absorption correction: analytical (*CrysAlis PRO*; Oxford Diffraction, 2009)	*h* = −32→18
*T*_min_ = 0.444, *T*_max_ = 1.000	*k* = −6→7
5100 measured reflections	*l* = −19→21

### Refinement

**Table:**

Refinement on *F*^2^	Primary atom site location: structure-invariant direct methods
Least-squares matrix: full	Secondary atom site location: difference Fourier map
*R*[*F*^2^ > 2σ(*F*^2^)] = 0.032	Hydrogen site location: inferred from neighbouring sites
*wR*(*F*^2^) = 0.068	H atoms treated by a mixture of independent and constrained refinement
*S* = 1.06	*w* = 1/[σ^2^(*F*_o_^2^) + (0.0213*P*)^2^] where *P* = (*F*_o_^2^ + 2*F*_c_^2^)/3
2633 reflections	(Δ/σ)_max_ < 0.001
151 parameters	Δρ_max_ = 0.55 e Å^−^^3^
0 restraints	Δρ_min_ = −0.60 e Å^−^^3^

### Special details

**Table:**

Geometry. All esds (except the esd in the dihedral angle between two l.s. planes) are estimated using the full covariance matrix. The cell esds are taken into account individually in the estimation of esds in distances, angles and torsion angles; correlations between esds in cell parameters are only used when they are defined by crystal symmetry. An approximate (isotropic) treatment of cell esds is used for estimating esds involving l.s. planes.
Refinement. Refinement of F^2^ against ALL reflections. The weighted R-factor wR and goodness of fit S are based on F^2^, conventional R-factors R are based on F, with F set to zero for negative F^2^. The threshold expression of F^2^ > 2sigma(F^2^) is used only for calculating R-factors(gt) etc. and is not relevant to the choice of reflections for refinement. R-factors based on F^2^ are statistically about twice as large as those based on F, and R- factors based on ALL data will be even larger.

### Fractional atomic coordinates and isotropic or equivalent isotropic displacement parameters (Å^2^)

**Table:**

	*x*	*y*	*z*	*U*_iso_*/*U*_eq_	
Br1	0.418223 (9)	−0.36757 (5)	0.230618 (14)	0.01800 (9)	
O1	0.33754 (6)	−0.2340 (4)	0.04409 (11)	0.0205 (4)	
O2	0.09326 (6)	0.3711 (4)	0.06453 (11)	0.0219 (4)	
O3	0.12126 (7)	0.6341 (3)	0.15486 (11)	0.0224 (4)	
N1	0.34163 (8)	0.0629 (4)	0.13870 (13)	0.0177 (5)	
N2	0.12933 (8)	0.4612 (4)	0.11152 (12)	0.0175 (5)	
C1	0.44903 (10)	−0.3344 (5)	0.05532 (15)	0.0205 (6)	
H1A	0.4520	−0.2507	0.0008	0.031*	
H1B	0.4251	−0.4681	0.0461	0.031*	
H1C	0.4860	−0.3844	0.0756	0.031*	
C2	0.42421 (9)	−0.1793 (5)	0.12266 (14)	0.0156 (5)	
C3	0.46064 (9)	0.0238 (5)	0.14342 (16)	0.0209 (6)	
H3A	0.4986	−0.0283	0.1564	0.031*	
H3B	0.4465	0.1044	0.1937	0.031*	
H3C	0.4605	0.1275	0.0937	0.031*	
C4	0.36323 (9)	−0.1220 (5)	0.09765 (14)	0.0143 (5)	
C5	0.28757 (9)	0.1555 (5)	0.12844 (14)	0.0133 (5)	
C6	0.24391 (9)	0.0415 (5)	0.08499 (14)	0.0156 (5)	
H6	0.2497	−0.1040	0.0595	0.019*	
C7	0.19187 (9)	0.1446 (5)	0.07971 (14)	0.0154 (5)	
H7	0.1618	0.0700	0.0503	0.018*	
C8	0.18422 (9)	0.3543 (5)	0.11722 (14)	0.0135 (5)	
C9	0.22692 (9)	0.4702 (5)	0.16051 (14)	0.0158 (5)	
H9	0.2208	0.6157	0.1857	0.019*	
C10	0.27856 (9)	0.3680 (5)	0.16594 (14)	0.0150 (5)	
H10	0.3083	0.4438	0.1957	0.018*	
H1N	0.3624 (11)	0.132 (5)	0.1742 (17)	0.028 (8)*	

### Atomic displacement parameters (Å^2^)

**Table:**

	*U*^11^	*U*^22^	*U*^33^	*U*^12^	*U*^13^	*U*^23^
Br1	0.01677 (13)	0.01963 (16)	0.01755 (13)	0.00024 (10)	−0.00059 (9)	0.00090 (10)
O1	0.0181 (8)	0.0222 (12)	0.0211 (9)	0.0011 (8)	−0.0017 (7)	−0.0079 (8)
O2	0.0143 (8)	0.0292 (13)	0.0218 (9)	0.0013 (8)	−0.0029 (7)	0.0000 (8)
O3	0.0204 (9)	0.0223 (12)	0.0247 (9)	0.0072 (8)	0.0027 (7)	−0.0027 (9)
N1	0.0135 (10)	0.0205 (14)	0.0187 (11)	0.0023 (9)	−0.0038 (8)	−0.0075 (10)
N2	0.0183 (10)	0.0190 (14)	0.0152 (10)	0.0039 (9)	0.0026 (8)	0.0028 (9)
C1	0.0180 (12)	0.0222 (17)	0.0214 (12)	0.0066 (11)	0.0030 (10)	−0.0025 (11)
C2	0.0151 (11)	0.0158 (15)	0.0159 (11)	0.0025 (10)	0.0004 (9)	−0.0007 (10)
C3	0.0141 (11)	0.0220 (17)	0.0265 (13)	0.0001 (11)	0.0008 (10)	0.0007 (12)
C4	0.0166 (11)	0.0144 (14)	0.0122 (11)	−0.0001 (10)	0.0032 (9)	0.0011 (10)
C5	0.0127 (11)	0.0165 (15)	0.0109 (10)	−0.0002 (10)	0.0020 (8)	0.0010 (10)
C6	0.0176 (11)	0.0145 (15)	0.0148 (11)	0.0015 (10)	0.0005 (9)	−0.0023 (10)
C7	0.0121 (11)	0.0187 (16)	0.0152 (11)	−0.0004 (10)	−0.0013 (9)	0.0010 (10)
C8	0.0119 (11)	0.0170 (15)	0.0117 (11)	0.0030 (10)	0.0024 (8)	0.0034 (10)
C9	0.0200 (12)	0.0120 (14)	0.0155 (11)	0.0010 (10)	0.0030 (9)	−0.0018 (10)
C10	0.0143 (11)	0.0177 (15)	0.0130 (11)	−0.0018 (10)	−0.0001 (8)	−0.0016 (10)

### Geometric parameters (Å, °)

**Table:**

Br1—C2	2.010 (2)	C3—H3A	0.9800
O1—C4	1.211 (3)	C3—H3B	0.9800
O2—N2	1.234 (3)	C3—H3C	0.9800
O3—N2	1.232 (3)	C5—C10	1.392 (4)
N1—C4	1.366 (3)	C5—C6	1.400 (3)
N1—C5	1.416 (3)	C6—C7	1.393 (3)
N1—H1N	0.84 (3)	C6—H6	0.9500
N2—C8	1.465 (3)	C7—C8	1.373 (4)
C1—C2	1.519 (3)	C7—H7	0.9500
C1—H1A	0.9800	C8—C9	1.387 (3)
C1—H1B	0.9800	C9—C10	1.382 (3)
C1—H1C	0.9800	C9—H9	0.9500
C2—C3	1.506 (4)	C10—H10	0.9500
C2—C4	1.546 (3)		
			
C4—N1—C5	127.9 (2)	H3B—C3—H3C	109.5
C4—N1—H1N	117 (2)	O1—C4—N1	123.5 (2)
C5—N1—H1N	115 (2)	O1—C4—C2	121.0 (2)
O3—N2—O2	123.4 (2)	N1—C4—C2	115.4 (2)
O3—N2—C8	118.4 (2)	C10—C5—C6	120.1 (2)
O2—N2—C8	118.2 (2)	C10—C5—N1	116.8 (2)
C2—C1—H1A	109.5	C6—C5—N1	123.2 (2)
C2—C1—H1B	109.5	C7—C6—C5	119.0 (2)
H1A—C1—H1B	109.5	C7—C6—H6	120.5
C2—C1—H1C	109.5	C5—C6—H6	120.5
H1A—C1—H1C	109.5	C8—C7—C6	119.7 (2)
H1B—C1—H1C	109.5	C8—C7—H7	120.1
C3—C2—C1	112.2 (2)	C6—C7—H7	120.1
C3—C2—C4	115.2 (2)	C7—C8—C9	122.2 (2)
C1—C2—C4	110.53 (19)	C7—C8—N2	119.3 (2)
C3—C2—Br1	108.18 (15)	C9—C8—N2	118.5 (2)
C1—C2—Br1	106.45 (18)	C10—C9—C8	118.2 (2)
C4—C2—Br1	103.51 (14)	C10—C9—H9	120.9
C2—C3—H3A	109.5	C8—C9—H9	120.9
C2—C3—H3B	109.5	C9—C10—C5	120.9 (2)
H3A—C3—H3B	109.5	C9—C10—H10	119.6
C2—C3—H3C	109.5	C5—C10—H10	119.6
H3A—C3—H3C	109.5		
			
C5—N1—C4—O1	0.7 (4)	C5—C6—C7—C8	−0.3 (3)
C5—N1—C4—C2	179.5 (2)	C6—C7—C8—C9	0.3 (4)
C3—C2—C4—O1	145.0 (2)	C6—C7—C8—N2	−179.9 (2)
C1—C2—C4—O1	16.6 (3)	O3—N2—C8—C7	170.6 (2)
Br1—C2—C4—O1	−97.1 (2)	O2—N2—C8—C7	−8.3 (3)
C3—C2—C4—N1	−33.9 (3)	O3—N2—C8—C9	−9.6 (3)
C1—C2—C4—N1	−162.3 (2)	O2—N2—C8—C9	171.6 (2)
Br1—C2—C4—N1	84.0 (2)	C7—C8—C9—C10	−0.4 (3)
C4—N1—C5—C10	−168.9 (2)	N2—C8—C9—C10	179.8 (2)
C4—N1—C5—C6	12.7 (4)	C8—C9—C10—C5	0.5 (3)
C10—C5—C6—C7	0.4 (3)	C6—C5—C10—C9	−0.5 (3)
N1—C5—C6—C7	178.7 (2)	N1—C5—C10—C9	−179.0 (2)

### Hydrogen-bond geometry (Å, °)

**Table:**

*D*—H···*A*	*D*—H	H···*A*	*D*···*A*	*D*—H···*A*
C6—H6···O1	0.95	2.27	2.862 (3)	120
C7—H7···O1^i^	0.95	2.45	3.139 (3)	129
N1—H1n···O3^ii^	0.84 (3)	2.66 (3)	3.316 (3)	136 (2)
C10—H10···Br1^iii^	0.95	2.91	3.812 (2)	160

Symmetry codes:  (i) −*x*+1/2, −*y*−1/2, −*z*; (ii) −*x*+1/2, *y*−1/2, −*z*+1/2; (iii) *x*, *y*+1, *z*.
